# Spontaneous adrenal hemorrhage in a newborn: a case report

**DOI:** 10.11604/pamj.2024.49.39.44725

**Published:** 2024-10-13

**Authors:** Jay Lodhia, Sophie Sikobizahora, Stephen Gondwe, Rune Nathaniel Philemon

**Affiliations:** 1 Department of General Surgery, Kilimanjaro Christian Medical Centre, P.O Box 3010, Moshi, Tanzania,; 2 Kilimanjaro Christian Medical University College, Faculty of Medicine, P.O Box 2240, Moshi, Tanzania,; 3 Department of Pediatrics and Child Health, Kilimanjaro Christian Medical Centre, P.O Box 3010, Moshi, Tanzania,; 4 Department of Radiology, Kilimanjaro Christian Medical Centre, P.O Box 3010, Moshi, Tanzania

**Keywords:** Adrenal hemorrhage, adrenal insufficiency, ultrasonography, case report

## Abstract

Neonatal adrenal hemorrhage is a rare condition with various risk factors in the pediatric population. Adrenal hemorrhage commonly affects the right side in about 70% of the cases and bilateral in about 10%. It is usually asymptomatic but can cause adrenal insufficiency and sudden death. Neonatal adrenal hemorrhage should be considered even when there are no risk factors, as it can occur spontaneously with unspecific clinical presentations. Ultrasonography can confirm diagnosis, a relatively cheap and safe modality, especially for resource-limited settings. In this case report, we present a two-day-old with progressive scrotal hematoma and inguinal ecchymosis with no risk factors. Magnetic resonance imaging confirmed right adrenal hemorrhage; however, the newborn deteriorated fast, reaching mortality within 24 hours of admission. This case illustrates the importance of recognizing the condition to allow for an evidenced approach, which may include conservative waiting, as unwarranted intervention can have negative outcomes.

## Introduction

The adrenal gland is vulnerable to mechanical compression in newborns due to its relatively large size and increased vascularity [1]. The high vascularity of the adrenal glands means its capillaries can easily rupture when traumatized or when they experience sudden rises in pressure [2]. The vulnerability of the adrenal vascular system is made worse by the high concentrations of catecholamines and epinephrine, which can drastically alter the pressures in these vessels [2]. Bleeding into the adrenal glands can be the result of direct trauma to the gland (often presenting as unilateral bleeding), but could also stem from various systemic causes such as infections, coagulopathies, or even complications of other pathologies such as adrenal tumors. Systemic causes will often present with bilateral bleeding [2]. Of recent, there have been reports linking neonatal adrenal hemorrhage (NAH) to coronavirus (COVID-19) infections [2,3].

Epidemiologically, NAH is a rare condition with an incidence of 0.2 - 0.55% [1]. Neonatal adrenal hemorrhage is commonly seen in term infants and mainly affects males [4]. Any factor that potentially predisposes the infant to birth trauma, such as macrosomia, and any factor that increases the risk of birth asphyxia potentially increases the risk of NAH as well [5]. We report a case of a two-day-old newborn with a progressive scrotal hematoma and inguinal ecchymosis that was confirmed by MRI to be right adrenal hemorrhage, despite the child having no known risk factors for NAH.

## Patient and observation

**Patient information:** a 2-day-old male baby was referred to our center from a peripheral hospital due to gradual abdominal distention and progressive scrotal swelling associated with discoloration extending to the left inguinal region. This was accompanied by gradual progressive difficulty in breathing to the extent that the baby was unable to breastfeed. The mother denied fevers and blue discoloration of the skin. The pregnancy had been uneventful, and the baby was born at term with a good APGAR score. Imaging was not possible at the facility where the child was delivered hence the referral.

**Figure 1:**
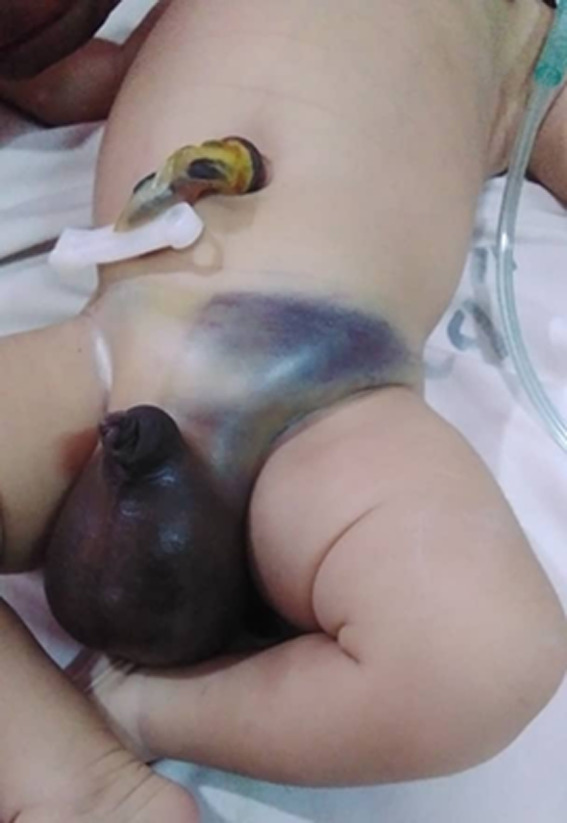
left inguinal ecchymosis with massive scrotal hematoma

**Clinical findings:** on examination, the baby was pale, afebrile, and had a respiratory rate of 72 breaths per minute. The abdomen was distended with ecchymosis around the left inguinal area and had a hematocele (Figure 1).

**Timeline of the current episode:** the presenting symptoms were noted at birth and with the abdominal distension ecchymosis and scrotal swelling increasing with time.

**Figure 2:**
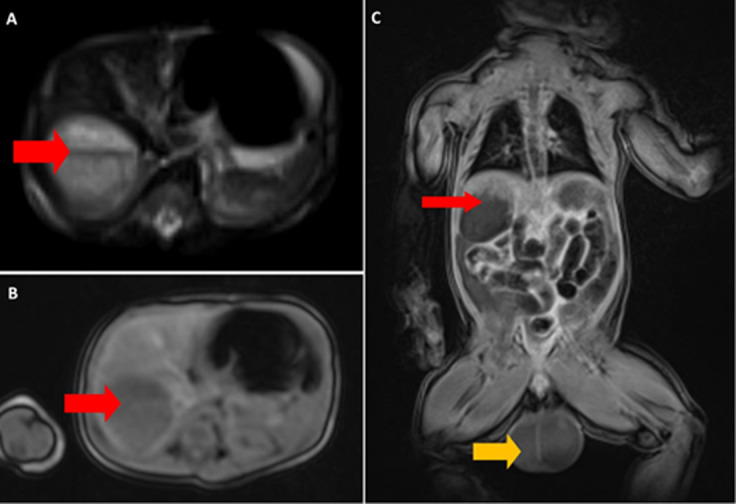
A, B) abdomen (axial view) showing a round lesion with a fluid-fluid level from the right suprarenal region measuring 3.8 x 3.9 x 3.4 cm, keeping with right adrenal bleeding (red); C) abdomen-pelvis (coronal view) showing right-sided adrenal hemorrhage (red) and scrotal hematoma (yellow)

**Diagnostic assessment:** his complete blood count revealed a WBC of 31 x 10^9^/L and hemoglobin of 6.9 g/dl with normal platelet count. The serum sodium was 121 mmol/L, and mild hyperkalemia of 5.9 mmol/L with elevated liver enzymes. Abdominal-pelvic ultrasound revealed right adrenal hemorrhage with bilateral scrotal hemorrhage. This was also confirmed by MRI (Figure 2).

**Diagnosis:** with the clinical findings and the investigations, the patient was diagnosed to have spontaneous right adrenal hemorrhage with bilateral scrotal hematoma and severe anemia. A differential of disseminated intravascular coagulopathy and multiorgan failure was also entertained.

**Therapeutic interventions:** the baby was kept on oxygen support, intramuscular vitamin K, intravenous antibiotics, and fluids. The baby was transfused with 80 ml of whole blood.

**Follow-up and outcome of interventions:** the baby continued to deteriorate rapidly and succumbed within 24 hours of arrival. The cause of death was presumed to be from a massive hemorrhage and adrenal insufficiency.

**Patient perspective:** the parents of the newborn expressed their gratitude to the team of doctors and nurses for their dedication and efforts in providing the best possible care for their child. Despite the initial challenges and concerns, they were appreciative of the team's commitment to ensuring a positive outcome for their newborn.

**Informed consent:** written informed consent was obtained from the patient´s mother to publish this case report; additionally, accompanying images have been censored to ensure that the patient cannot be identified. A copy of the consent is available on record.

## Discussion

This case highlights the critical importance of maintaining a high index of suspicion and ensuring early intervention in cases of neonatal adrenal hemorrhage (NAH), as not all patients will present with clear risk factors. In resource-limited settings, the absence of essential diagnostic tools, such as basic ultrasound, can contribute to delayed presentation and diagnosis, ultimately impacting the patient's prognosis. This underscores the need for improved access to healthcare resources and timely diagnostic capabilities in these settings.

Adrenal hemorrhage was first described in 1863 by Canton. Early diagnosis is difficult due to its unspecific clinical presentation; hence, the majority of the cases are diagnosed during surgery or autopsy [6]. Each adrenal gland is fed by three arteries and drained by one vein. This means there is a large capillary network forming within the gland. Any occlusion to the vein can easily trigger back pressure into the capillaries, causing them to hemorrhage. The high concentration of epinephrine around the vein means it is vulnerable to platelet aggregation and vasoconstriction [2]. The right adrenal gland is more often affected as it drains directly into the inferior vena cava, thus being affected by changes in venous pressure more than the left. It also sits higher up and can therefore be squeezed between the ribs and the liver [5].

The clinical presentation of NAH largely depends on the extent of the bleeding and the associated damage to the adrenal glands [6]. Small bleeds often go unnoticed and may be picked up as an incidental finding. More substantial bleeds can present with non-specific features such as abdominal masses during physical examination and jaundice due to the breaking down of the hematoma [5]. When the hemorrhage ruptures the capsule of the adrenal gland, blood can descend through the retroperitoneal space into the scrotum and present as a scrotal hematoma [7]. The resulting scrotal swelling can mimic a myriad of other scrotal conditions, and thus, a high index of suspicion is necessary whenever scrotal hemorrhage is encountered in the neonate [8,9]. As with any severe hemorrhage, shock is a possible complication, and some patients do present with hypovolemic shock [7].

The adrenal cortices can sustain up to 90% damage before signs of adrenal insufficiency appear. It is thus rare to see adrenal insufficiency, usually when there is extensive bilateral hemorrhage [2,6]. Adrenal insufficiency results in electrolyte imbalances and can spiral quickly into shock and sudden death [10]. In a patient with feeding difficulties and hemorrhage, it is challenging to ascertain what is causing the symptoms and deterioration without a high index of suspicion. With our patient, it appears that the hemorrhage had ruptured the adrenal capsule, resulting in bleeding into the retroperitoneal space and descending into the scrotum. This, we believe, is what gave the presentation of severe anemia and scrotal swelling.

Imaging is key to the diagnosis of NAH, with ultrasonography being the mainstay. Other imaging modalities, such as magnetic resonance imaging, can provide additional information [2,7]. Additional investigations to help with the assessment of potential complications are also necessary. At the basic, these should include a full blood count to assess the extent of anemia. Electrolytes, particularly sodium and potassium, will help point you toward adrenal insufficiency, as will the serum glucose levels and ketones [7,10]. As coagulopathies and neonatal sepsis can predispose to NAH, workup for these should also be part of the laboratory assessment.

Management of NAH mainly takes a conservative, non-surgical approach. This is largely due to the great ability of the adrenal glands to self-repair [5]. The complications, such as adrenal insufficiency, can pose a life-threatening emergency and should thus be managed promptly [5]. In patients where the conservative approach is failing, as in those not responding to transfusion due to massive bleeding, vascular interventions such as transarterial embolization are recommended over exploratory laparotomy [2]. Resolution of the hemorrhage can take up to nine months, and it is advised to monitor the patient with regular ultrasounds until the hemorrhage has resolved [4,5].

## Conclusion

Neonatal adrenal hemorrhage is a rare but critical condition with clinical presentations that can easily be mistaken for other disorders. Early and accurate diagnosis requires a high index of suspicion. The primary focus in managing NAH should be on vigilant monitoring for complications and providing stabilization to the patient, allowing for the natural healing of the adrenal glands. Surgical intervention should be reserved for cases where conservative management fails. Prompt and appropriate management is crucial to improving outcomes and preventing mortality in affected neonates.
